# The mediating role of learning motivation in the relationship among perceived stress and emotional regulation among Saudi nursing students in clinical practice

**DOI:** 10.1186/s12912-024-01893-1

**Published:** 2024-04-16

**Authors:** Bander Saad Albagawi, Yasir S. Alsalamah, Abdulqadir J. Nashwan, Rakan Mansuor AL Rawili, Lisa A. Babkair, Sara A. Alkharji, Thamer Alslamah, Mirna Fawaz

**Affiliations:** 1 Medical Surgical Department, College of Nursing, University of Hail, Hail City, Saudi Arabia; 2 Department of Nursing, Mental Health Hospital, Qassim Health Cluster, Qassim, Buraydah, Saudi Arabia; 3 Faculty of Health Science, Department of Nursing, University of Pretoria, Pretoria, South Africa; 4 Nursing Department, Hamad Medical Corporation, Doha, P.O. Box 3050, Qatar; 5 General Directorate of Health Affairs in Aljouf, General Director of Academic and Training in Aljouf Health, Cluster Aljouf, Aljouf, Skaka, Saudi Arabia; 6 Training Department, Indiana Kokomo University, Indiana, Kokomo, USA; 7 Faculty of Nursing, King AbdulAziz University, 21589 Jeddah, Saudi Arabia; 8 Department of Mental Health, King Saud University Medical City, Nursing Affairs, Riyadh City, Saudi Arabia; 9 Department of Public Health, College of Public Health and Health Informatics, Qassim University, Al Bukairiyah, Saudi Arabia; 10 College of Health Sciences, American University of the Middle East, Egaila, Kuwait

**Keywords:** Emotional regulation, Stress, Motivation, Nursing education, Nursing students

## Abstract

**Background:**

Nursing students often face high levels of stress due to demanding responsibilities during clinical placement. Emotional regulation, the ability to manage and regulate one’s emotions effectively, is crucial for nursing students in dealing with stress and maintaining their overall well-being. Additionally, learning motivation plays a vital role in students’ engagement and academic success. The current investigation aimed at studying the link that exists among stress, learning motivation, and emotional regulation among Saudi undergraduate nursing students. The study also aimed at investigating the sequential mediating effects that motivation might perform in this association.

**Methods:**

A quantitative cross-sectional methodology was used in the present research, which recruited 367 Saudi undergraduate nursing students.

**Results:**

The results of the ANOVA showed that the level of perceived stress was linearly and negatively correlated with emotional regulation and motivation. Upon conducting structural equation modeling, significant direct and indirect effect pathways were identified between perceived stress, emotional regulation, and motivation, while only indirect pathways were identified between perceived stress and emotional regulation.

**Conclusions:**

This study provides evidence of the mediating role of learning motivation in the relationship between perceived stress and emotional regulation among Saudi nursing students. The results highlight the negative impact of stress on emotional regulation and learning motivation and emphasize the importance of addressing motivational factors in interventions aimed at enhancing emotional regulation among nursing students.

## Background

Student nurses must complete intensive nursing skill training in addition to receiving nursing schooling in order to fulfill the demands of clinical opportunities. A wide range of procedures are included in the practice of nursing, including collecting specimens, taking measurements, conducting sterile procedures, giving treatments and IV infusions, saving severely ill individuals, providing reassurance to patients, and providing emotional support [1]. All nursing staff members must possess these essential capacities. The level of care, medical condition, security of patients, and the caliber of therapy are all directly impacted by the appropriate method. As a result, nursing students’ training must include instruction in nursing competencies [2]. Practical educational programs are typically used to undertake nursing competency improvement. There exists a strong correlation between the characteristics and feelings that students display during hands-on training and their educational performance [3]. A German scholar, Pekrun, came up with the notion of academic emotions while researching how people learn [4].

Emotional regulation refers to a person’s capacity to respond to stress as a coping mechanism that controls a variety of processes, including emotional, behavioral, cognitive, physiological, and environmental processes. It has gained significant attention as a potential contributing component to issues with social adaptation. It has been discovered that emotional control in nursing students reduces anxiety and enhances their ability to function as nurses [5]. Emotions are significant since they serve as messages to the person, informing them that certain incidents or circumstances are sufficiently significant to warrant a reaction. In an educational setting, these might be instances, such as receiving feedback, where students perceive themselves as moving toward or away from their objectives. However, these cues may be ignored or misinterpreted; feelings may be helpful or detrimental to reaching objectives [6]. The process of regulating emotions relies on the wants and ambitions of a person. The goal of emotional regulation techniques is to change an emotion’s composition, strength, communication, or endurance [7].

Within the mechanistic framework of emotion regulation, two commonly studied strategies are cognitive reappraisal and suppression [7]. Cognitive reappraisal is considered an adaptive emotion regulation strategy as it allows individuals to modify their emotional responses by reframing the meaning of a situation in a way that reduces its emotional intensity. By employing cognitive reappraisal, individuals can shift their focus toward more positive aspects of a situation or reinterpret its significance, thereby regulating their emotional reactions effectively [8]. This strategy has been associated with various positive outcomes, including enhanced emotional well-being, reduced negative affect, and improved interpersonal functioning [9]. On the other hand, suppression, as an emotion regulation strategy, involves inhibiting the outward expression of emotions without necessarily changing their underlying emotional experience. While suppression may temporarily reduce the visible signs of emotional expression, it does not address the underlying emotional experience and can lead to negative consequences in the long run [9]. Research has shown that suppression can be associated with increased physiological arousal, decreased positive affect, and impaired interpersonal interactions [10]. Therefore, cognitive reappraisal is generally regarded as a more adaptive strategy compared to suppression.

Stress has a well-known impact on students’ conduct and psychological well-being. Dangerous environmental cues are the source of stress. Perceived emotions are generated after people analyze and assess the alarming signals using their mental abilities. These emotions are known as “perceived stress” [11]. Prior research has discovered a strong inverse relationship among a person’s pleasant feelings and perceived stress [12]. A different study found that learners reported stress was inversely connected with their joyous feelings [13]. It is possible to deduce that there are variables that operate as mediators between emotional conduct and perceived stress. Examining the effects of these variables may highlight individual variations.

Motivation to learn is the factor that not only commences education but also keeps it going and guides it. According to Urhahne and Wijnia [14], motivation to learn is an intrinsic drive and mental capacity that positively impacts the education of students. The kind and level of motivation to learn has a meaningful impact on the orientation, pace, and outcome of the learning process. Research indicates a strong relationship between learning motivation and stress, where pleasant learning emotions are positively impacted by motivation to learn [15].

The Resource Preservation Theoretical Model proposes a framework for understanding the processes underlying emotion regulation and its impact on individuals’ cognitive, emotional, and behavioral outcomes [16, 17]. This model suggests that individuals engage in emotion regulation strategies to preserve their personal and environmental resources, thereby facilitating adaptation and well-being. In the context of the present study, the variables of perceived stress, emotional regulation, and learning motivation can be integrated within the framework of this model. Perceived stress represents the individual’s appraisal of the demands and challenges they experience in their clinical practice. Higher levels of perceived stress may indicate a greater need for effective emotion regulation strategies to preserve personal and environmental resources. Learning motivation, as a variable in this study, can be considered as a potential mediator within the Resource Preservation Theoretical Model. Learning motivation reflects the individual’s appraisal of the value and importance of their educational experience. It is plausible that higher levels of learning motivation may lead nursing students to actively engage in adaptive emotion regulation strategies, such as cognitive reappraisal, as a means to preserve personal and environmental resources in the pursuit of successful learning outcomes [17].

Previous research has established a significant association between perceived stress and emotional regulation. Studies have shown that higher levels of perceived stress are often linked to difficulties in regulating emotions effectively [18, 19]. Individuals experiencing elevated levels of perceived stress may exhibit maladaptive emotional regulation strategies, such as suppression or avoidance, which can further exacerbate the negative impact of stress on their well-being [20]. As for the relationship between stress and motivation it has been demonstrated to be a paradox in the literature, where evidence is not conclusive [21]. In addition, the relationship between emotional regulation and motivation has only been the subject of theoretical work [22] and very few empirical studies [23], especially when considering stress. There is a dearth of studies specifically examining these relationships among Saudi nursing students in the context of clinical practice. Given the unique challenges faced by nursing students during their clinical training, such as high workload, exposure to critical situations, and emotional demands, investigating the mediating role of learning motivation can provide valuable insights into the mechanisms underlying the relationship between perceived stress and emotional regulation in this specific population. Addressing this gap can inform the development of targeted interventions and support programs to enhance nursing students’ learning motivation and promote their emotional well-being in the clinical setting. Therefore, the aim of this study was to investigate the mediating role of learning motivation in the relationship between perceived stress and emotional regulation among nursing students. We hypothesized that motivation would mediate the association between perceived stress and emotional regulation.

## Methods

### Research Design

This research is a subset of a bigger project that was carried out in Saudi Arabia involving students studying nursing. A quantitative cross-sectional research design was employed in the research in order to look for any relationships between the factors being studied. Cross-sectional designs help assess connections between variables at a certain point in time, despite its restriction on the establishment of causal linkages among variables.

### Sample

This investigation involved nursing learners from King Saud University in Saudi Arabia who were enrolled in the Bachelor of Nursing Program (BSN). The students represented a variety of academic levels, excluding first-year students. Convenience sampling provided the greatest opportunity for the enrollment of students. Students who were proficient in English maintained continuous enrollment in the BSN program and had no record of suspension or dismissal were among those who satisfied the study’s qualifying standards. Most importantly, to be eligible to participate the student should have finished at least the first clinical placement in the first year. Students who had previously enrolled in a technical nursing diploma program changed their major and later enrolled in the BSN program, were moved into the program, or had a record of social or mental issues were not included to take part in the study. A power analysis was conducted to determine the right number of participants. Based on an estimated population that comprised 600 nursing students, a 95% confidence level, and a 5% margin of error, 235 students were needed in the sample. However, because structural equation modeling is used in this work to create a mediation model, a ratio of 15:1 was used to calculate the necessary sample size, which was based on an 80% power level and a moderate effect size of 0.3. Consequently, there was a need for an additional 105 students, meaning that 340 students would be the total sample size needed to sufficiently power the study and find an effect.

### Setting

One of the major colleges in the nation and the very first to be formed in Saudi Arabia is King Saud University. The intended university provides a four-year nursing program with a required internship to prepare students for the workforce. A variety of learning possibilities are offered by the course of study to students, such as instruction in the classroom, high-fidelity simulations, problem-based learning through video, team-based learning, and more. Furthermore, students are required to undertake clinical practice at the affiliated hospital during their study period. In their clinical training students are assigned one patient per clinical day if they were practicing in a critical care area and 3–4 patients per clinical day if they were assigned to a regular floor. This is determined by the intended learning outcomes of the respective courses they are enrolled in, which specify certain nursing tasks to fulfill during clinical rotations.

## Procedure

### Recruitment and data collection

The director of the nursing department at King Saud University was contacted and asked for a sample frame. They provided the investigators with a list of current nursing students. The list included the students’ university emails. 410 students received an email invitation to participate in this study. The email included the primary investigator’s contact details, a request for a consent form, and detailed study information. Those that replied with a consent form filled out were included. An 89% response rate was achieved by the 367 students out of the 410 who were asked to take part. Clear and concise communication about the study’s objectives and confidentiality of responses helped motivate students to take part. In addition, convenience sampling often relies on making participation as easy and convenient as possible for potential participants. This included factors such as flexible timing, convenient locations through which they could complete the questionnaire, and minimal burden on participants’ schedules. Moreover, the researchers made sure that the questionnaire was user-friendly, easily understandable, and not excessively time-consuming. The researchers also implemented a reminder system which was beneficial to prompt potential participants who have not yet responded. The students cited personal circumstances as the reason they chose not to participate in the study. A data-collecting period extending from September 2022 to April 2023 was utilized.

### Instrumentation

#### Sociodemographic questionnaire

A demographic data sheet was used to collect information about the student’s gender, age, and academic level.

#### Emotional regulation questionnaire (ERQ)

The current study assessed participants’ emotion regulation skills using the emotion regulation questionnaire (ERQ) [9]. The ERQ is a 10-item questionnaire that assesses how much people rely on cognitive reappraisal and suppression strategies to control their emotional states. Cognitive reappraisal refers to efforts to control emotional experience by changing one’s interpretation of internal and external cues. In contrast, suppression refers to efforts to prevent behavioral responses that result from emotional states. Both terms are used in process models of emotion regulation (Gross, 2015). Participants reported their level of agreement with each of the presented items using a 7-point Likert-type scale (1 = strongly disagree, 7 = strongly agree). Prior research has established the factorial and convergent validity of the instrument when applied to university students. Further, reliability analyses indicated that the reappraisal (Cronbach’s a = 0.84, McDonald’s & = 0.85) and suppression (Cronbach’s a = 0.78, McDonald’s & = 0.78) subscales of the ERQ demonstrated acceptable levels of internal consistency in the current investigation. In this study, the Cronbach alpha was 0.90. Numerous studies have provided evidence supporting the construct validity of the ERQ. For example, Gross and John conducted a series of studies demonstrating the convergent validity of the ERQ by examining its associations with other measures of emotion regulation, affect, and interpersonal outcomes [9]. Additionally, studies have investigated the factorial structure of the ERQ, supporting the presence of two distinct factors corresponding to cognitive reappraisal and suppression, where also Gyurak et al. (2011) examined the discriminant validity of the ERQ by comparing it to measures of implicit emotion regulation [24]..

#### Perceived stress scale (PSS)

We assessed undergraduate students’ perceptions of stress using the 10-item perceived stress scale (PSS) [25]. The PSS measures comprehensive perceived stress experienced across the past 30 days on a 5-point scale (0– never, 1 = almost never, 2 = once in a while, 3 = often, 4 = very often). Six of the 10 items were worded and scored in the non-reversed direction (i.e., “How often have you felt that you were unable to control the important things in your life”). Four of the 10 items were worded and scored in the reversed direction (i.e., “How often have you felt that things were going your way”). Total scores range from 0 to 40. The PSS was shown to demonstrate acceptable levels of internal consistency in the current examination (Cronbach’s a = 0.82, McDonald’s & = 0.81). Numerous studies have provided evidence supporting the construct validity of the PSS. For example, Cohen et al. (1983) conducted a validation study of the PSS and found that higher scores on the scale were associated with self-reported stress-related symptoms and measures of psychological distress [26]. The PSS has also demonstrated discriminant validity by showing differential relationships with other constructs. For instance, studies have found that the PSS correlates positively with measures of psychological distress, anxiety, and depression while showing weaker or non-significant correlations with constructs such as social support and self-esteem [27].

#### Motivated strategies for learning questionnaire (MSLQ)

The Motivated Learning Strategies Questionnaire (MLSQ) was utilized in this study to assess and evaluate the motivational factors influencing learning strategies among participants. The MLSQ, originally developed by Pintrich and De Groot [28] is a well-established instrument designed to measure students’ motivational beliefs and self-regulated learning strategies across various academic contexts. Participants respond to items on a Likert-type scale from 1 to 7, indicating the extent of their agreement or disagreement with statements related to each subscale. The higher the total score, the higher the motivation for learning. The Cronbach alpha for this study was 0.92. Studies have examined the factorial structure of the MSLQ in different educational contexts. While there are variations in the number of factors identified across studies, the MSLQ has consistently demonstrated a multidimensional structure, indicating that it measures multiple underlying constructs related to motivation and self-regulated learning [29, 30]. The MSLQ has also demonstrated discriminant validity by showing differential relationships with other constructs. For instance, studies have found that the MSLQ subscales correlate differently with constructs such as self-efficacy, test anxiety, and academic achievement, supporting its ability to distinguish between different aspects of motivation and learning [29, 30].

### Data analysis

The data was analyzed using Statistical Package for Social Science (SPSS) version 26. Frequencies were used to describe the characteristics of the sample. Means, standard deviations, and confidence intervals were computed to explore the central tendency and spread of the data. Independent t-tests were used to explore the association between study variables and gender. ANOVA was used to determine if there is an association between academic level and study variables, as well as, perceived stress level and study variables. AMOS was used to carry out structure equation modeling (SEM) to test direct and indirect effects among study variables. The positioning of the variables and the hypothesized direct and indirect paths are justified by the fact that the model being tested is based on both empirical and theoretical considerations, which are used to explore possible relationships between the variables.

### Ethical considerations

The researchers granted the approval from the Qassim University’s Research and Ethics Committee (Name and Number: ECO-R-191). All ethical considerations were applied according to the International Declaration of Helsinki’s principles and guidelines, where the students were informed about all details of the study before recruitment and were not forced to be inducted. The researchers are not engaged in any instructional activities with the participating students. No disadvantages were reported to students who did not participate, and written informed consent was obtained.

## Results

### Participants’ characteristics

The study sample was made up of 264 (71.9%) male students and 103 (28.1%) female students. The majority of the students in the sample were in their second and third year of study, where the mean age of the participants was 21.11 ± 1.63 years (Table 1).

**Table 1:** Participants characteristics

Category	N	%
Gender		
Male	264	71.9
Female	103	28.1
Academic Level		
Semester 1	2	0.5
Semester 2	3	0.8
Semester 3	65	17.7
Semester 4	62	16.9
Semester 5	90	24.5
Semester 6	22	6.0
Semester 7	74	20.2
Semester 8	17	4.6
Internship	32	8.7
Age (M ± SD)	21.11 ± 1.63	

### Descriptive statistics of study variables

The mean perceived stress score of the participating students was 23.60 ± 7.73, which is within the range for moderate perceived stress level. The mean motivation score was 136.60 ± 34.93, and that of emotional regulation was 43.23 ± 14.00 (Table 2). By examining the Q-Q plots, it seems that the data is approximately normally distributed with minor skewness to the left. The confidence intervals had minimal standard errors. Descriptive statistics showed that the vast majority (58%) of the students reported moderate stress levels, while 103 (28.1%) reported a high stress level. The mean scores of motivation and emotional regulation were examined by the level of perceived stress. The results showed that data was more normally distributed among students reporting moderate stress. Despite the histograms and normality curves in the other stress groups having minor skewness to the left, the Q-Q plots show indications of normality. This is expected considering the unequal groups, where the results of the descriptive statistics indicate unequal variances. The descriptive statistics also indicate how mean scores change according to ascending levels of perceived stress (Table 2).

**Table 2:** Distribution of scores of study variables

	Mean		SD		SE		95% CI (LB, UB)
Perceived Stress	23.60		7.73		0.40		22.81, 24.40
Motivation Total	136.6		34.93		1.82		133.00, 140.17
Emotional Regulation	43.23		14.00		0.73		41.79, 44.67
Perceived Stress	N		%		-		-
Low	51		13.9		-		-
Moderate	213		58		-		-
High	103		28.1		-		-
Perceived Stress	Low		Moderate		High		**-**
	Mean ± SD	Variance	Mean ± SD	Variance	Mean ± SD	Variance	
Motivation	159.80 ± 36.11	1304.47	123.95 ± 35.07	1230.20	116.90 + 55.51	3081.6	**-**
Emotional Regulation	54.43 ± 11.52	132.72	42.41 ± 9.45	89.37	24.00 ± 12.17	148.08	**-**

LB: Lower Bound

UB: Upper Bound

### Association between study variables and participant characteristics

An independent T-test was carried out to determine if there was an association between gender and the study variables. The results of Levene’s test showed equal variances for all variables. The test did not show any significant differences in means according to gender, where the recorded *p*-values were > 0.05, except for emotional regulation, where males recorded a higher mean than females (*p* = 0.04). An ANOVA was carried out to determine if there is an association between academic level and the study variables. The homogeneity of variance test showed no significant difference. The results of the ANOVA test showed no significant differences in the tested variables according to academic level (Table 3).

**Table 3:** Difference in total scores according to gender and academic level

		Mean	SD	Test statistic	95% CI	*P*-value
Perceived Stress	Male	23.76	7.71	0.59	-1.23449, 2.30304	0.55
	Female	23.22	7.83			
Motivation	Male	137.95	36.05	1.13	-3.31617, 12.28902	0.26
	Female	133.46	32.12			
Emotional Regulation	Male	44.16	14.13	2.04	0.11807, 6.49138	**0.04**
	Female	40.85	13.47			
		Sum of Squares	df	Mean Square	F	*P*-value
Motivation	Between Groups	18905.907	9	2700.844	1.60	0.13
	Within Groups	427577.57	358	1683.376		
	Total	446483.48	367			
Perceived stress	Between Groups	345.225	9	43.153	0.72	0.68
	Within Groups	21554.85	358	60.209		
	Total	21900.07	367			
Emotional Regulation	Between Groups	2022.17	9	252.772	1.30	0.24
	Within Groups	69803.14	358	194.98		
	Total	71825.31	367			

Note: significant *p*-values are bold

A bivariate Pearson correlation was carried out between the total scores of the study variables and age. The results showed that there was a positive linear statistically significant relationship of varying strength between all perceived stress (*p* = 0.00), motivation (*p* = 0.018), emotional regulation (*p* = 0.00), and age. The strongest correlation was noted between age and perceived stress (Table 4).

**Table 4:** Correlations between study variables and age

		Perceived Stress	Motivation	Emotional Regulation
Age	R-value	0.225	0.124	0.195
	*P*-value	**0.000**	**0.018**	**0.00**
	N	367	367	367

Note: significant *p*-values are bold

### Association between study variables

An ANOVA test was conducted to determine if there is an association between perceived stress levels and motivation and emotional regulation. The homogeneity of variance test showed no significant difference. The results showed that the motivation (*p* = 0.00) and emotional regulation (*p* = 0.00) scores were statistically significantly different according to perceived stress levels. A post-hoc Bonferroni test was conducted and showed a significant difference in emotional regulation scores between all stress levels (*p* = 0.00), and a significant difference in motivation scores between low and high (*p* = 0.00), as well as moderate and high (*p* = 0.00) (Table 5).

**Table 5:** Difference in total scores according to perceived stress level

			Sum of Squares	df	F		Sig.
**Motivation**		Between Groups	75599.396	9	26.96		**0.00**
		Within Groups	337895.141	358			
		Total	413494.537	367			
**Emotional Regulation**		Between Groups	22295.112	9	96.55		**0.00**
		Within Groups	36136.821	358			
		Total	58431.934	367			
**Motivation**	Mean Difference (I-J)	Std. Error	*P*-value	95% Confidence Interval			
				Lower Bound		Upper Bound	
Low-Moderate	7.06	8.95	1.00	14.53		28.65	
Moderate-High	35.84	5.16	**0.00**	23.41		48.27	
Low-High	42.90	9.31	**0.00**	20.47		65.33	
**Emotional Regulation**	Mean Difference (I-J)	Std. Error	*P*-value	95% Confidence Interval			
				Lower Bound		Lower Bound	
Low-Moderate	18.50	2.26	**0.00**	13.07		23.93	
Moderate-High	11.86	1.30	**0.00**	8.73		14.98	
Low-High	30.36	2.34	**0.00**	24.72		35.99	

Note: significant *p*-values are bold

A Pearson’s bi-variate correlational analysis was carried out to determine if there is a linear association between the study variables. The results showed that there was a moderate positive statistically significant linear association between motivation and emotional regulation (*r* = 0.57, *p* = 0.00). A weak negative statistically significant linear association was detected between perceived stress and motivation (*r*=-0.437, *p* = 0.00) and a moderate negative statistically significant linear association was detected between perceived stress and emotional regulation (*r*=-0.698, *p* = 0.00) (Table 6).

**Table 6:** Correlation between study variables

		Motivation	Perceived stress	Emotional Regulation
Motivation	Pearson Correlation	1	− 0.437^**^	0.571^**^
	Sig. (2-tailed)		**0.000**	**0.000**
	N	367.00	367.00	367.00
Perceived stress	Pearson Correlation	− 0.437^**^	1	− 0.698^**^
	Sig. (2-tailed)	**0.000**		**0.000**
	N	367.00	367.00	367.00
Emotional Regulation	Pearson Correlation	0.571^**^	− 0.698^**^	1
	Sig. (2-tailed)	**0.000**	**0.000**	
	N	367.00	367.00	367.00

Note: significant *p*-values are bold

### SEM and mediation analysis

Based on the previous results multiple structural equation models were conducted to identify how the association between the study variables operates. The results showed that significant pathways exist between the study variables (*p* = 0.00), where a bi-directional path might exist between motivation and emotional regulation (Table 7).

**Table 7:** Standardized path coefficients

	Standardized coefficients	Standard Error	Lower Bound	Upper Bound	*P*-value
Perceived Stress → Motivation	-2.47	0.33	-1.82	-3.12	**0.00**
Perceived Stress → Emotional Regulation	-1.27	0.074	-1.13	-1.42	**0.00**
Motivation → Emotional Regulation	1.79	0.16	1.463	2.109	**0.00**
Emotional Regulation → Motivation	1.65	0.17	1.89	3.03	**0.00**

Note: significant *p*-values are bold

The results further demonstrated that perceived stress had a significant direct, indirect, and total effect on motivation, yet no significant direct effect on emotional regulation. However, a significant direct effect was observed between motivation and emotional regulation (*p* = 0.00). Thus, this indicates that the association between perceived stress and emotional regulation is mediated by motivation and the association between motivation and emotional regulation could be further moderated by perceived stress and other unexplored factors (Table 8).

**Table 8:** Direct and Indirect Effect coefficients and significance

	Direct Effect	Indirect Effect	Total Effect
Perceived Stress → Motivation	-1.85 (**0.00**)	-0.63 (**0.00**)	-2.47 (**0.00**)
Perceived Stress → Emotional Regulation	-0.43 (0.05)	-0.82 (**0.00**)	-1.24 (**0.02**)
Motivation → Emotional Regulation	1.42 (**0.00**)	0.27 (**0.00**)	1.69 (**0.00**)
Emotional Regulation → Motivation	1.32 (**0.00**)	0.43 (**0.00**)	1.64 (**0.00**)

Note: significant *p*-values are bold

## Discussion

The present study aimed to investigate the mediating role of learning motivation in the relationship between perceived stress and emotional regulation among Saudi nursing students. Consistent with previous research [31, 32], our results showed a negative correlation between perceived stress and emotional regulation. This finding suggests that higher levels of stress are associated with poorer emotional regulation abilities among nursing students. These findings are in line with the transactional model of stress and coping, which posits that stress can hinder individuals’ ability to effectively regulate their emotions [33]. The demands and challenges faced by nursing students, such as heavy workloads, clinical responsibilities, and academic pressure, may contribute to elevated stress levels, which in turn can compromise their emotional regulation skills. Scholars’ perspectives and focus increasingly evolved from unfavorable to pleasant feelings due to the impact of positive psychology. The relationship between stress and favorable emotions has been the subject of recent studies [34]. This finding can also be attributed to the culture of the students, where under stress young adults usually tend to suppress undesirable emotions rather than communicate them with others. In other cultures, it could have been a different case depending on the dynamics of interpersonal relationships. Thus, further investigation into this aspect could further our knowledge.

In addition, in the present investigation, emotional regulation and motivation to learn among nursing students were positively associated, where motivation was a significant predictor of the latter. This conclusion, which centered on the fundamental ideas of motivational theoretical terms, was in line with the findings of previous research conducted by Chen, Huang [35]. Better educational accomplishment and more favorable educational emotions are the results of students’ increased commitment and motivation to learn [36]. Additionally, those who are highly motivated to learn are more inclined to look for proactive coping strategies that work, which helps them feel less distressed [37].

Additionally, among Saudi nursing students, our study showed a negative link between experienced stress and motivation to learn. This result is consistent with other studies that have shown the negative effects of stress on students’ motivation [38]. This may be described by the cognitive appraisal process, where individuals perceive stresses as threats, resulting in a decline in their motivation levels [39]. High levels of stress might make nursing students less motivated to participate in class activities and work toward their academic objectives.

Most importantly, consistent with previous work [11], our study revealed the mediating effect of learning motivation between perceived stress and emotional regulation among Saudi nursing students. Specifically, higher levels of stress were associated with reduced learning motivation, which, in turn, led to poorer emotional regulation skills. This finding highlights the importance of addressing motivational factors in interventions aimed at enhancing emotional regulation among nursing students. According to earlier research, highly driven people typically retain pleasant emotions and an optimistic outlook in addition to seeing stress and life’s challenges from an optimistic viewpoint [40]. People with greater drive were more successful in embracing favorable and hopeful attitudes and high stress amid circumstances and at getting more social assistance therefore lessening their sensation of anxiety and diminished power. These abilities helped to foster and sustain positive emotions in the learning environment. Continuous development is necessary to build learning motivation, which is impacted by a variety of factors including awareness of oneself, family history, social context, and educational surroundings [41]. Thus, throughout nursing students’ clinical practice, universities, family members, and healthcare facilities should focus more on motivation to learn. Scholars and instructors should develop realistic strategies for addressing perceived stresses in certain learning environments, such as student nurses’ anxiety and diminished power in clinical settings, and refrain from stifling beneficial academic feelings. Moreover, since the most significant determinant of emotional regulation is academic drive, it is advised that instructors focus on fostering students’ enthusiasm in nursing clinical practice in order to enhance their ability to regulate emotions. This will help the learners’ educational achievement and their nursing skills.

### Limitations and recommendations

One of the factors that limit our inquiry is our design. It is not possible to determine a causal link among the variables under study due to the cross-sectional design. Cross-sectional designs cannot determine whether the observed associations are causal or if they exist due to reverse causality, they do not capture the timing of events or changes in variables, and they do not account for the potential effects of maturation or time-related factors. Therefore, in order to get a deeper comprehension of the relationships between these factors, more studies on this subject should investigate and assess mediational frameworks using a longitudinal approach.

Another disadvantage is the multicollinear influence of using self-reporting for variable measurement. In order to reduce subjectivity and get more information about possible modifications in the parameter interactions, future research may use execution or performance methodologies instead of SEM, which was employed in this work to offset these effects.

An additional issue is the convenience sampling design. This may have resulted in a sampling bias and a non-representative sample that does not accurately reflect the larger population of nursing students or the diversity within that population, thus limiting the ability to make broader inferences or generalize the findings to other populations or settings. Further research could be done using data points from various educational institutions to generalize the findings.

Future research could involve conducting comparative studies to examine potential differences in the relationships between the variables across different contexts, educational systems, or cultural settings. This can provide valuable insights into the contextual factors that may influence the observed associations. Moreover, employing qualitative research methods, such as interviews or focus groups, can be valuable in gaining a deeper understanding of the experiences, perceptions, and contextual factors related to the variables under investigation.

## Conclusion

The findings of this research show a substantial negative correlation between the participants’ motivation to learn and emotional management during clinical placement on one hand and participants’ reported stress on the other hand. Motivation to learn and reported stress were shown to be important precursors of emotional regulation. The investigation also demonstrated the critical role that motivation to learn had in regulating the relationship among felt stress and emotional regulation among student nurses, as in mitigating the negative effects of stress. Effectively enhancing learners’ motivation to learn might make them feel less stressed and more optimistic. Nurses who experience positive emotions during their education would possibly be better able to develop their abilities and become more competent nurses.

Nursing educators can use our results to develop strategies and interventions aimed at reducing stress levels among students. This may include implementing stress management programs, providing mentorship and support systems, and promoting self-care practices. In addition, our results call on nursing educators to enhance students’ intrinsic motivation for learning. Strategies such as incorporating engaging teaching methods, providing opportunities for autonomy and choice, and establishing clear learning goals can help foster students’ motivation to learn and regulate their emotions effectively. Nursing leadership and policymakers can also play a crucial role in allocating resources and implementing policies that prioritize the well-being of nurses and nursing students alike. This includes advocating for adequate high-quality nursing programs, staffing levels at nursing schools and hospitals, safe work environments, and comprehensive support systems for students during their clinical practice. By recognizing the unique challenges faced by nursing students and implementing supportive policies, legislators can contribute to the overall quality of nursing education and the well-being of future healthcare professionals.

## Data Availability

The datasets used and/or analyzed during the current study are available from the corresponding author on reasonable request.

## Declarations

The researchers granted the approval from the Qassim University’s Research and Ethics Committee (Name and Number: ECO-R-191). All ethical considerations were applied according to the International Declaration of Helsinki’s principles and guidelines, where the students were informed about all details of the study before recruitment and were not forced to be inducted. No disadvantages were reported to students who did not participate, and written informed consent was obtained.

## Abbreviations

ERQ: Emotional Regulation Questionnaire
PSS: Perceived Stress Scale
SEM: Structural Equation Modeling
MLSQ: Motivated Learning Strategies Questionnaire
